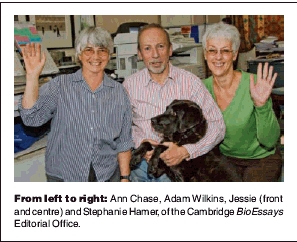# Farewell from the Editor and the staff of the Cambridge office of *BioEssays*

**DOI:** 10.1002/bies.20853

**Published:** 2008

**Authors:** Adam S Wilkins

**Affiliations:** Dept. of Zoology, University of Cambridge, Downing St.Cambridge CB2 3EJ, U.K. E-mail: wilkins316@btinternet.com

With this double issue of *BioEssays*, for November and December, the last for 2008, I lay down my red pen and retire from the editorship of this journal. I am turning it over to the new Editor, Dr Andrew Moore, formerly of EMBO, with all my best wishes to him for a long, successful and enjoyable term at the helm of *BioEssays*.

This note is, thus, a farewell to “my” authors and the readers of this journal but it is also a thank you note to these groups, whose interest in and support of *BioEssays* have (obviously) been essential for its existence and survival. I feel more than general gratitude to these overlapping constituencies, however: one of the great pleasures of the job has been the chance to get to meet and/or correspond, about all sorts of things, with so many of our authors and readers.

Farewells, whether spoken or written, are inevitably somewhat self–centred and, often, somewhat sentimental. To avoid or, at least reduce, these elements, I would like to devote this space to something other than the business of saying goodbye, namely a short history of How *BioEssays* Got to Be the Way It Is. We have tried, and, I hope, succeeded in making this a distinctive journal and the story of how it acquired its identity may be of interest. It is a proper evolutionary tale, not a “just so” story. In fact, it turns out to be, in part, a story of co–evolution.

This history begins, naturally enough, with the founding editor of *BioEssays*, Dr William J. Whelan, of the University of Miami School of Medicine. Bill had previously been the founding editor of *Trends in Biochemical Sciences*, which began publication in 1976. Though review articles as such and the long reviews of the *Annual Reviews* series had existed well before 1976, TIBS was the first modern review journal, one carrying short reviews designed to inform readers about recent developments in particular subjects. Bill is thus, in a sense, the Father of Review Journals. (He also originated the idea of the poster session, and is thus also the Father of Poster Sessions, but that is another story.) Bill left TIBS in 1979 but, of course, continued to read it and follow its course. By the early 1980s, he had begun to feel that TIBS was not doing full justice to the emerging area of molecular genetics and that a new review journal devoted to genetics would be appropriate—none existed at the time. As incredible as it will seem to many readers of this piece who are in their early 40s or younger, the world was not then awash in review journals. In fact, I believe, there was only TIBS for short reviews. Thus, in 1983, Bill began to canvass the possibilities for a new review journal centred on genetics with potential publishers and soon reached an agreement with Cambridge University Press (CUP).

What should the new journal be called? Bill and CUP decided that, naturally enough, it should be called *Trends in Genetics*. Unbeknownst to them, however, Elsevier Publishers were well along in planning their *Trends* series at this point, including a *Trends in Genetics*. Elsevier soon got wind of CUP's plans and rapidly communicated its displeasure. In fact, they indicated that legal action would almost certainly follow if CUP went ahead with naming its journal *Trends in Genetics*. The Elsevier position was that they had an exclusive legal right to any title beginning with the words “Trends in”. This “advice” required a rapid rethink on the part of CUP, and Bill soon came up, in late 1983, with the name *BioEssays*. Thus, the title was born.

It was about this point that I entered the story. Having resigned from a university position that I was unhappy with, I had moved myself and my family to Cambridge, England, to finish a book I was then writing (on developmental genetics) and to look for funds for a research position at the University, in Michael Ashburner's lab where I had previously completed a sabbatical. Just a few months after returning to Cambridge, however, I got a call from the journals' department head at CUP, who asked: might I be interested in becoming the managing editor of a new genetics journal that was then starting? Perhaps, I felt, this would be worth trying; I could always go back to hunting for research money if I did not like it. CUP had previously hired someone for this post but she had backed out at the last minute and with only six months to go before the start of publication, and very little material in the pipeline, they needed someone desperately. An unemployed American academic, who knew some genetics, might do just fine. I passed the interview, which was informal and brief, and was hired.

Bill had already started commissioning articles but, early in 1984, we mostly just had the new name, *BioEssays*. It was apparent to us, however, that this name liberated us from the narrow constraints of the original mission of the journal, namely to cover advances in molecular genetics. We could, in principle, cover *all* biology. This possibility seemed much more exciting and pleased us both. It was clear that the old disciplinary boundaries—genetics, cell biology, biochemistry, developmental biology—were rapidly dissolving and that a truly integrative, multi-disciplinary review journal might be the appropriate sort of journal to recognize that fact.

With an initial volume size of only 48 pages, however, there was the question of how one wouldmake such a broad–ranging journal of sufficient interest to enough people to turn them into subscribers. Individuals who only read articles in their subject area would hardly find enough of interest in any one issue of that size.

One way to spark such interest would be to have a large section of “feature articles”, namely articles of great diversity and greater liveliness than standard review articles. They would be easy to read and, if done well, would have appeal beyond particular subject boundaries. Bill and I discussed possibilities and he also soon set up an editorial advisory group, the “Friends of *BioEssays*”, consisting of about half a dozen distinguished Cambridge scientists, in part to brainstorm about this. I remember, in particular, however, that the idea for the “My Favourite Cell” column—devoted to new and interesting model cell systems – originated with Bill. This eventually blossomed into the whole “My Favourite…” series. (The term “my favourite…” now crops up regularly in talks all over the world and I believe that this reflects the influence of our series.) But more than variety was needed: we would also have to do everything possible to make the articles readable and interesting. In doing so, we could (we hoped) tempt people to read and thus learn about subjects outside their subject area—*BioEssays* would, potentially, become a broad educational vehicle. But to help the authors with the task of writing for an audience beyond their own specialties, we would have to understand the material fairly well ourselves. My chief responsibility was the features section and I soon found that I had to educate myself across a whole range of subjects, in order to edit the material with any proficiency. In effect, I soon became a *de facto* student again, which suited me to a tee. I had been hired primarily as the nuts-and-bolts guy to make sure that the journal's production ran smoothly but I now had some real editorial responsibility plus a license to learn as much biology as possible. Though our early coverage was heavily weighted toward molecular genetics, cell biology and biochemistry, we were soon branching out into other fields, as a start toward justifying the “Bio” in *BioEssays*.

In late 1989, after the journal was well–established, Bill left *BioEssays* to become editor–in–chief of the *FASEB Journal*. Through a complex series of events, which I will not detail here, the journal moved to a new company, the Company of Biologists, Ltd. (the COB, publishers of *Development*, the *Journal of Cell Science*, and the *Journal of Experimental Biology*) and I became the Editor of *BioEssays*. Under the COB, the journal got a face lift and a corresponding boost in production values (better paper, a glossy cover, and a more attractive type-face) and I set about to improve the readability of the articles and to further expand the range of coverage, particularly into the area of evolutionary biology, which had always been a strong interest of mine. Two critically important colleagues in this new phase of the journal's life were Dr Kermit L. Carraway (a colleague of Bill's at the University of Miami School of Medicine) and Dr Robert T. Johnson (then of the Department of Zoology, the University of Cambridge, and later Director of the MRC's Mary Lyon Centre at Harwell).

But while working away at the progressive evolution of *BioEssays* into an even better journal—at least that was the goal—I began to notice that I, too, was changing. One change I have already mentioned. I had metamorphosed back into being a student, in fact a perpetual student. Indeed, on visiting my alma mater, Reed College, for my 40^th^(!!) anniversary class reunion in 2005, I realised that during my employment with *BioEssays*, I had effectively turned back into being a Reed student. Reed takes intelligent, idiosyncratic kids and exposes them to a huge range of subjects, demands lots of written work (indeed, essays), vast amounts of reading, and a never-ending series of deadlines for pieces of work. There are no formal grades but lots of informal evaluation. In the end, Reed turns out young adults who are, in general, far more knowledgeable, more curious about the world as a whole, and probably even more idiosyncratic than when they entered. I reckon that, thanks to *BioEssays*, I have managed to remain a Reed student for a total of 28 years (though only four were spent at the college itself), surely a record even for a tertiary institution famous for its number of perpetual students.

But my personal co–evolution with *BioEssays* went beyond this. If you want to work constructively with authors to improve further their manuscripts, you have to use finesse and diplomacy, which is also simply the decent thing to do. Every article involves a lot of work by its author(s) and those efforts should be acknowledged, since the editor often has to ask the author(s) to put in even more work, in revision. Such requests should be done courteously and in a spirit that is clearly intended to be helpful. In effect, to be an editor, at least of a review journal, one has to acquire the skills of a diplomat. In addition, in the course of all my contacts with people, especially on my travels in connection with work, I began to lose some of the social awkwardness all too typical of academics, especially male academics. I found myself more at ease in social situations and confident that I was handling them well or, at least, better than I had before. Thus, thanks to *BioEssays*, I discovered my “inner extrovert” (a mind–boggling concept, when one considers it).

It has been a terrific job in all respects, although a demanding one. While it will be good to leave its ceaseless pressures behind, there is a part of me that will always miss it—though, at the same time I am eager to get on with the next stage of my life, which will continue to be involved with biology in numerous ways (thinking and writing about it, doing free-lance editing, teaching, etc.) It has also been gratifying to have received so much praise for my work over the years from numerous people though, at the same time, there is the nagging feeling that, after all, I was only trying to do the work conscientiously and perhaps one should not be praised for just doing one's job. But I would like to say that whatever I have put into the work, the job would have been impossible without the support and active help of many people. *BioEssays* has been a truly co–operative effort involving numerous individuals. Some of the key figures (Bill Whelan, Bob Johnson, Kermit Carraway,“The Friends of *BioEssays*”) are acknowledged above and I would like to stress how much I appreciate their contributions and support. But there are four others, as well, whom I want to thank. First is Joe Ingram, my top manager at John Wiley & Sons for the 11 years that this journal has been with Wiley. (It moved from the Company of Biologists in 1997.) Joe has been friendly, sympathetic and helpful throughout; I could not have asked for a better “boss”.

And then, and most crucially, there have been three key assistants: Stephanie Hamer (my general assistant–secretary–office manager), Ann Chase (my assistant editor), and Eleanor Wick (Ann's daughter and Stephanie's predecessor). My job has been a complex one and, at times, has felt overwhelming. Without the highly competent, efficient and above all, friendly and steady support of Steph, Ann and Eleanor, I would have gone under long ago. I am deeply grateful to them that this did not happen.

And so, from the three of us at the Cambridge office, along with our invaluable canine support crew (my dog Jessie), we bid farewell to our readers and authors, with all best wishes to each of you for health, happiness and much enjoyment of your work.